# Illuminating the Spectrum: A Spectrophotometric Exploration of Beverage Effects on Hybrid and Nano-Hybrid Resin Composites: An In Vitro Study

**DOI:** 10.7759/cureus.116944

**Published:** 2026-09-25

**Authors:** Ahmad Albahoth, Abdulhakeem A Almutairi, Sabahat Ullah Khan Tareen, Shahzad Ali shah, Abdulaziz Waleed Alhazmi, Feras a Alajlan, Kadi S Alharbi

**Affiliations:** 1 Conservative Dental Sciences, College of Dentistry Qassim University, Buraidah, SAU; 2 Dentistry, College of Dentistry Qassim University, Buraidah, SAU

**Keywords:** beverages, color stability, discoloration, hybrid composite, nano-hybrid composite, resin composite, spectrophotometry

## Abstract

Resin composite restorations are widely used for their aesthetics and mechanical durability, yet their clinical longevity is often limited by discoloration and surface degradation caused by dietary chromogens and acidic beverages. This in vitro study aimed to evaluate and compare the color stability of a universal hybrid composite (Filtek Z250) and a nano-hybrid composite (Filtek Z350) after exposure to tea, coffee, and pomegranate juice, using distilled water as a control, quantified by spectrophotometry. Eighty standardized composite discs (10 mm × 2 mm) were fabricated (n=40/material), LED-cured per manufacturer instructions, and polished using a uniform multi-step protocol. Specimens were immersed individually in 10 mL of the assigned liquid and stored at 37°C and 100% relative humidity, with color measured at baseline and after one, seven, and 30 days using CIELAB parameters; color change was calculated as ΔE, and each specimen was measured three times to enhance reliability. Mean ΔE values increased with immersion time for both materials in all beverages, indicating time-dependent discoloration. Coffee produced the greatest staining (hybrid: one day 1.25, seven days 2.70, 30 days 4.70; nano-hybrid: 1.10, 2.10, 3.90), followed by tea (hybrid: 1.10, 2.50, 4.00; nano-hybrid: 0.95, 1.90, 3.20) and pomegranate juice (hybrid: 0.90, 1.80, 3.50; nano-hybrid: 0.85, 1.50, 3.00), whereas distilled water caused minimal change (hybrid: 0.20-0.40; nano-hybrid: 0.15-0.34). Across beverages and time points, the hybrid composite consistently showed higher ΔE than the nano-hybrid composite; however, the reported paired t-test p-values exceeded 0.05, suggesting no statistically significant differences under the stated threshold. Overall, commonly consumed beverages-particularly coffee-can meaningfully compromise composite color stability over time, and the lower discoloration observed for nano-hybrid resin supports its preferential use when staining exposure is anticipated, alongside patient counseling on dietary staining risks to preserve restoration aesthetics.

## Introduction

In restorative dentistry, resin composites are extensively utilized because of their excellent aesthetic properties and strong mechanical durability, which play a crucial role in enhancing patient satisfaction and achieving positive clinical results [1]. While resin composites have numerous advantages, their long-term clinical performance is frequently compromised by issues such as discoloration and surface deterioration. These problems are often attributed to the prolonged contact with dietary chromogens and acidic components found in many popular beverages [2,3]. Several beverages like coffee, tea, cola, and fruit juices have staining pigments and acidic components that can either infiltrate the resin matrix or interact with filler particles, causing undesirable changes in both the color and surface integrity of restorative materials [3-5]. Hybrid and nano-hybrid resin composites are differentiated by the size, shape, and distribution of their filler particles [6,7]. Hybrid composites contain a combination of macro- and micro-sized filler particles, whereas nano-hybrid composites utilize nano-sized fillers to improve polishability, aesthetics, and mechanical properties [6]. These compositional modifications can significantly affect the materials' staining susceptibility, water absorption, and surface roughness when exposed to different staining solutions and acidic environments [7,8].

Spectrophotometric analysis has become a reliable and balanced approach for quantitatively assessing color variations in dental materials [8,9]. By evaluating reflected light at specific wavelengths, spectrophotometers convey accurate colorimetric data that can categorize delicate discolorations not easily seen by the naked eye [9]. This process outclasses subjective visual assessments and guarantees consistent and standardized measurements, making it especially useful for comparative in vitro research [8,9,10]. This in vitro study was conducted to systematically assess and compare the impact of various commonly consumed beverages on the color stability of hybrid and nano-hybrid resin composites through spectrophotometric analysis. By replicating oral exposure conditions, the study seeks to offer clinically relevant insights into the performance and durability of materials, thereby aiding clinicians in selecting materials and advising patients on dietary choices to enhance the longevity and aesthetic results of resin composite restorations [1,2].

Aim

The purpose of this study is to evaluate and compare the color stability of hybrid and nano-hybrid composite restorative materials after exposing them to three beverages- tea, coffee, and pomegranate juice using spectrophotometry.

## Materials and methods

Specimen preparation

Hybrid and nano-hybrid resin composite samples were carefully crafted using precisely designed cylindrical molds with dimensions of 10 mm in diameter and 02 mm in thickness, ensuring consistency and reproducibility among all specimens (Table 1). The composite materials were methodically layered into the molds to avoid voids and promote uniform polymerization. A calibrated LED curing unit (SmartLite Pro Modular LED; Dentsply, Charlotte, North Carolina, USA) with a wavelength range of 450-480 nm was employed for polymerization, strictly following the manufacturer's specified curing times, light intensity, and protocols. After polymerization, the specimens were gently removed from the molds and underwent a standardized, multi-step polishing process. To minimize surface roughness, staining, and degradation, specimens were finished using a pressure-responsive abrasive disc (Enhance Finishing System; Dentsply, Charlotte, North Carolina, USA) modulated from firm to light pressure. Final superficial polishing refinement was achieved with a 1.0 μm aluminum oxide paste (Prisma Gloss Extra Fine; Dentsply, Charlotte, North Carolina, USA), producing a high-gloss surface matrix. For each resin composite, 40 disk specimens were made. Therefore, the total sample size was 80 (Figure 1).

**Table 1:** Composition of composite material (hybrid and nano-hybrid resin composites)

Material	Product	Category	Composition	Manufacturer
Resin-based Composites	Filtekz 350, A1 SHADE	Nano-hybrid resin composite	BIS-GMA, UDMA, BIS-EMA, PEGDMA, TEGDMA, surface-modified zirconia/silica (3 µ or less) Non-agglomerated /non-aggregated 20 nanometer surface - modified silicaparticles 68% by volume	3MESPE, St. Paul, MN, USA
	Filtekz 250, A1 SHADE	Universal hybrid Composite	BIS-GMA, UDMA, BIS EMA, zirconia/silica fillers (0.01-3.5 mm) 66% by volume	3M ESPE, St. Paul, MN, USA

**Figure 1:**
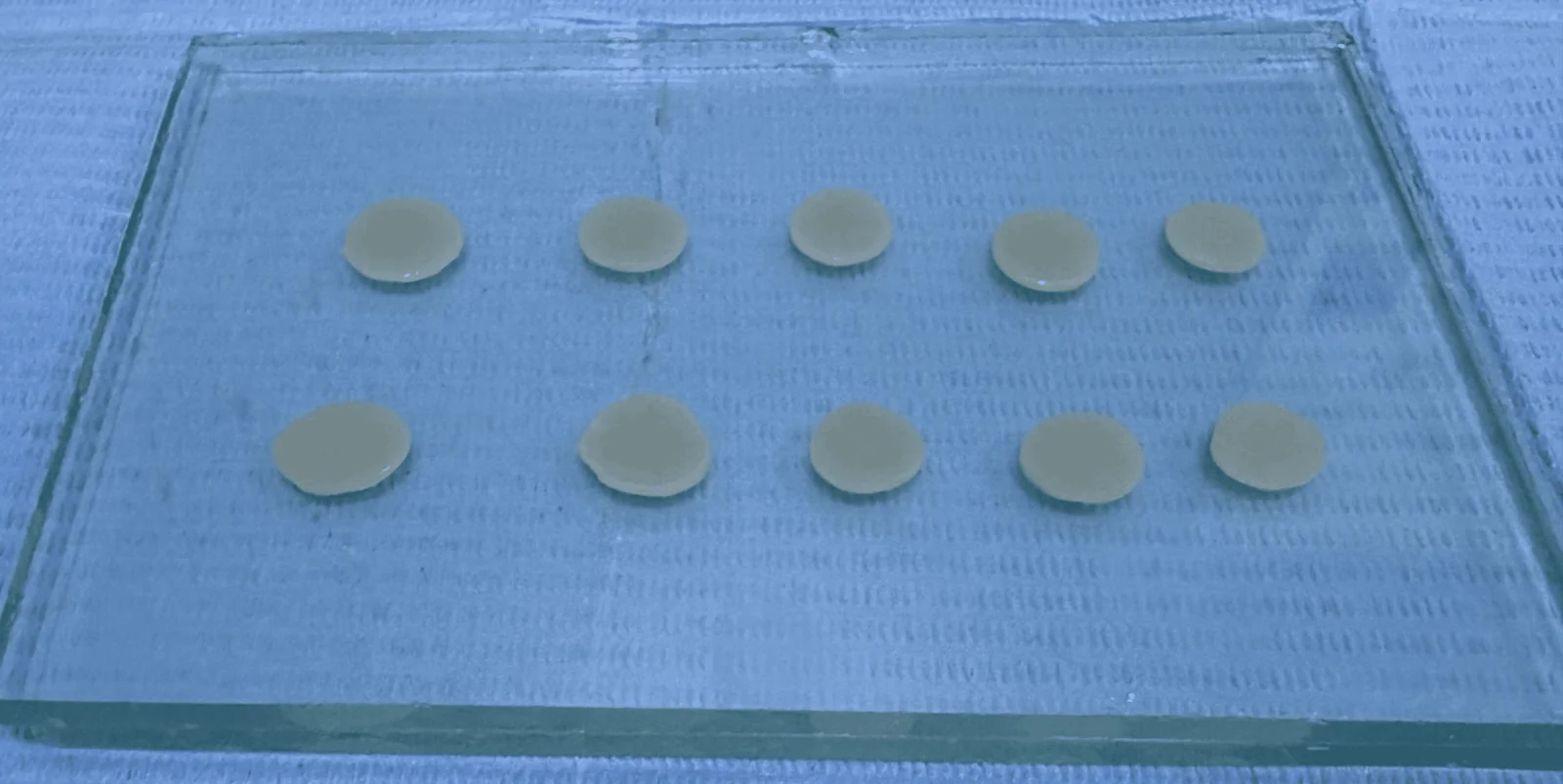
Composite disc made from hybrid and nano-hybrid composite

Beverage selection and immersion protocol

This research is based on four different immersion liquids: coffee (Nescafe Classic; Nestle, Vevey, Vaud, Switzerland), tea (Lipton Teas and Infusions; Amsterdam, Netherlands), and pomegranate juice (Almarai, Riyadh, Saudi Arabia), with distilled water used as a control. The samples were obtained from commercial sources. Each liquid has unique staining and acidic properties, resulting in surface wear and discoloration of dental materials. Coffee and tea are the most commonly consumed beverages with great staining capability, while pomegranate juice was selected for its strong acidic properties and intense pigmentation. Distilled water was used as a control. 

Each sample was immersed separately in a labeled glass container in 10 mL of the liquid at room temperature. The immersion durations were set at one, seven, and 30 days to assess both short-term and long-term impacts on color stability. To eliminate any buildup of beverage staining agent on the surface, it was rinsed with distilled water at room temperature. We replicated the temperature and moisture of an oral environment by maintaining the samples at 37°C and 100% relative humidity using a standard incubator and a humidified container setup. The CIELAB color space parameters - L (lightness), a (red-green axis), and b (yellow-blue axis) - were documented under a standardized CIE standard illuminant D65 setting and a 2-degree standard observer angle, capturing spectral reflectance across a visible spectrum range of 400-700 nm to prevent environmental ambient lighting bias. Further color assessments were conducted at intervals of one, seven, and 30 days after immersion to track color changes over time and evaluate the extent of discoloration. The color difference (ΔE) was determined using the formula ΔE = [(ΔL)² + (Δa)² + (Δb)²]¹/², offering a quantitative measure of discoloration compared to the initial values. This objective spectrophotometric method allowed for the detection of minor color variations that might not be visible to the naked eye, ensuring high sensitivity, precision, and consistency in evaluating the staining effects of various beverages on resin composites. Each sample was measured three times to reduce measurement error and improve reliability.

Statistical analysis

The colorimetric data collected were thoroughly analyzed using statistical methods to assess the significance and clinical importance of color variations between the hybrid and Nano-hybrid groups, considering different beverage exposures and immersion times. A paired t-test was used, and a threshold for statistical significance was established at p < 0.05. Statistical analyses were carried out using the validated and widely recognized software package SPSS 17 (IBM Inc., Armonk, New York, USA). For descriptive statistics, the mean and standard deviation were used. This precisely crafted and systematically robust approach enables a comprehensive and reproducible analysis of the impact of immersing hybrid and Nano-hybrid resin composites in coffee, tea, pomegranate juice, and distilled water for durations of one, seven, and 30 days on their color stability. By closely replicating clinical exposure conditions, the study design yields clinically pertinent data that can inform decisions regarding material selection, restorative dental procedures, and dietary guidance for patients, ultimately aiming to enhance the aesthetic longevity and functional resilience of resin composite restorations.

## Results

The results showed clear color changes among the two composites (Figures 2, 3). The overall maximum color change took place in hybrid resin composites, followed by nanocomposite resins. The coffee beverage subgroup showed more changes, as compared to other beverage subgroups at different durations in all the composite resins. This was followed by nano-hybrid composites in coffee beverage subgroups. The least color change took place in distilled water (Table 2). Time had an effect on the surface of both composites.

**Figure 2:**
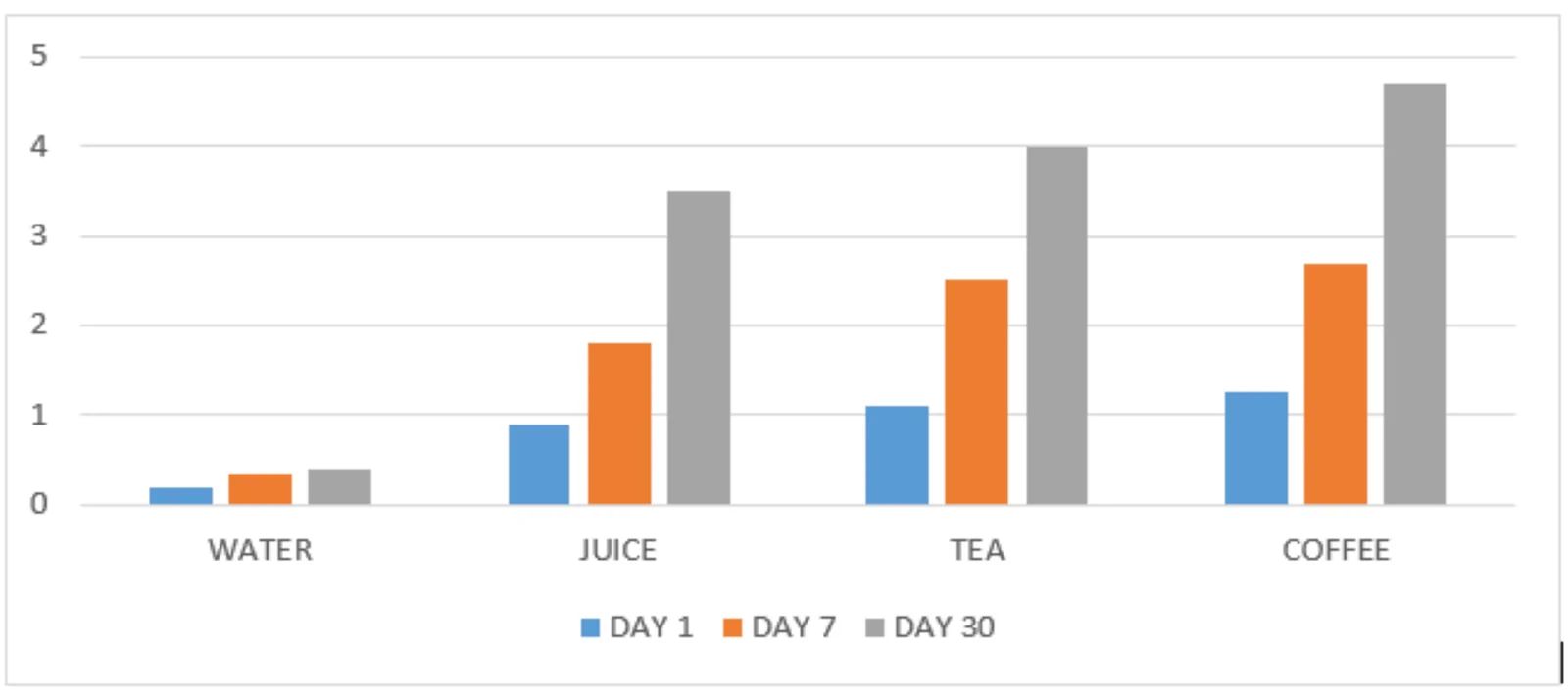
Graph showing mean ΔE values over time for hybrid composite in different beverages

**Figure 3:**
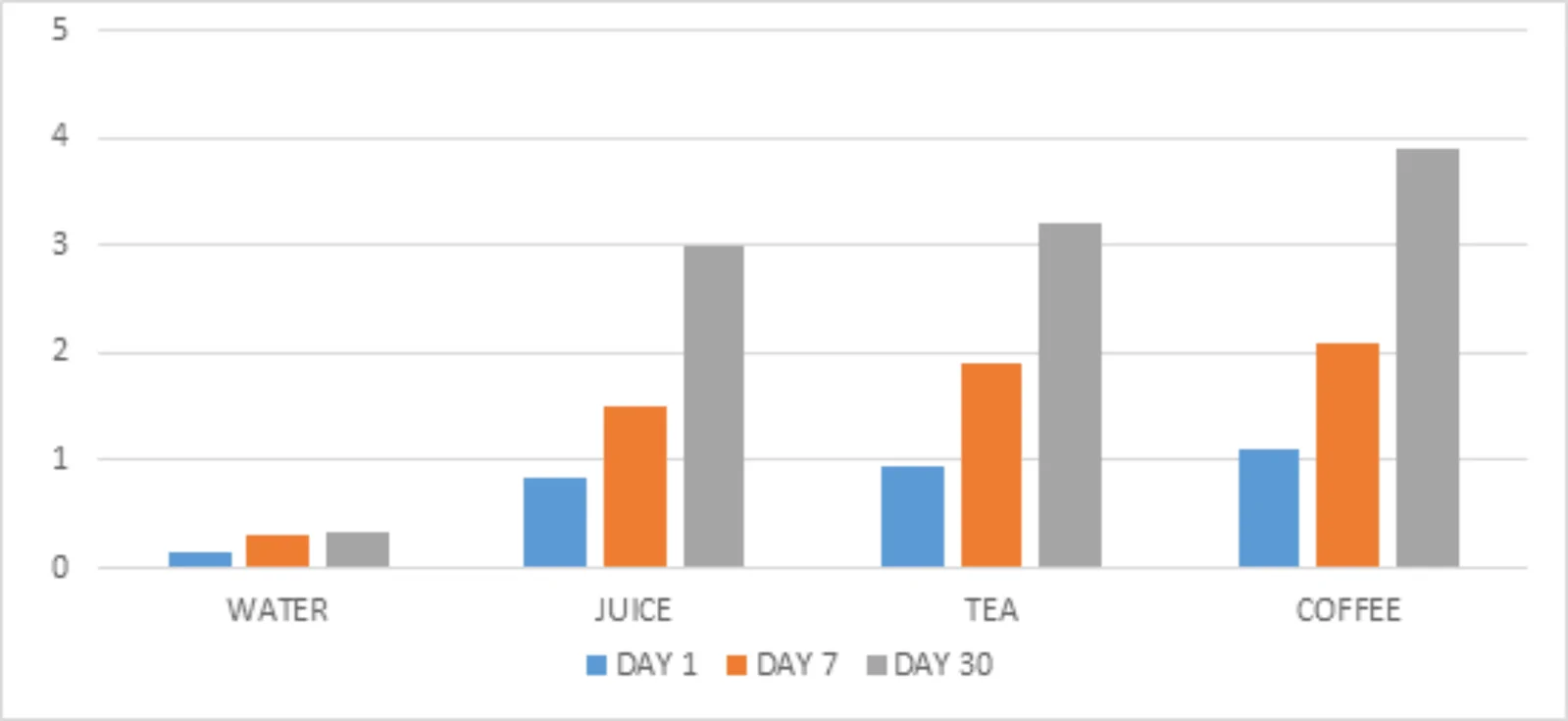
Graph showing mean ΔE values over time for nano-hybrid composite in different beverages

**Table 2:** Comparison of color changes mean ΔE values (p-value) for hybrid and nano-hybrid resin composites after immersion in different beverages over time

Beverage	Composite type	ΔE at 1 day	p-value	ΔE at 7 days	p-value	ΔE at 30 days	p-value
Coffee	Hybrid	1.25	0.15	2.70	0.30	4.70	0.40
	Nano-hybrid	1.10	0.12	2.10	0.25	3.90	0.35
Tea	Hybrid	1.10	0.10	2.50	0.28	4.00	0.38
	Nano-hybrid	0.95	0.11	1.90	0.22	3.20	0.30
Pomegranate juice	Hybrid	0.90	0.08	1.80	0.20	3.50	0.33
	Nano-hybrid	0.85	0.07	1.50	0.18	3.00	0.28
Distilled Water	Hybrid	0.20	0.05	0.35	0.07	0.40	0.10
	Nano-hybrid	0.15	0.04	0.30	0.06	0.34	0.08

## Discussion

The current research thoroughly evaluated the color stability of hybrid and nano-hybrid resin composites after being immersed in coffee, tea, pomegranate juice, and distilled water for periods of one, seven, and 30 days, using spectrophotometric analysis as an accurate and objective method to measure color changes [5,8]. The results clearly show that both types of composites undergo noticeable discoloration when exposed to chromogenic and acidic drinks, with the extent of color change increasing over time [3,5,11]. Among the beverages tested, coffee presented the highest staining potential, followed by tea and pomegranate juice, while distilled water, used as the control, had minimal effects, confirming its role as a baseline medium [5,9]. The more significant discoloration seen in hybrid composites compared to nano-hybrid composites across all beverage types and time intervals can be mainly attributed to differences in filler particle size, distribution, and resin matrix composition [6,7]. Hybrid composites typically consist of a varied mix of macro- and micro-sized filler particles, which may lead to increased surface roughness and micro voids, allowing for greater adsorption and penetration of staining agents [3,10]. In contrast, nano-hybrid composites contain nano-sized fillers that create a more uniform and densely packed microstructure, reducing surface irregularities and limiting the entry of chromogens, thus enhancing resistance to discoloration [6,11]. These findings are consistent with existing literature that emphasizes the superior polishability, smoother surface texture, and improved aesthetic durability of nano-hybrid composites compared to hybrid variants [6,7]. The observed gradual increase in ΔE values with longer immersion times reflects the cumulative and time-dependent nature of discoloration processes [2,5]. Chromogenic compounds found in coffee and tea, such as tannins and polyphenols, interact with both the resin matrix and filler particles, leading to surface adsorption and deeper stain penetration [4,5]. Although pomegranate juice has lower chromogenicity, its acidic nature may cause surface erosion of the composites, facilitating increased stain uptake and worsening discoloration [3,10]. This dual mechanism involving chemical staining and surface degradation highlights the multifactorial causes of beverage-induced color changes in resin composites [1,10].

Spectrophotometric analysis provided a highly sensitive and consistent method for detecting subtle color changes that might not be visible to the naked eye [8,9]. The statistically significant interactions between composite type, beverage, and immersion time highlight the intricate relationship between material properties and environmental factors influencing color stability [2,11]. These results highlight the importance of choosing restorative materials that resist staining, tailored to the dietary habits and lifestyle of individual patients [1,7]. Clinically, the superior color stability shown by nano-hybrid resin composites supports their preferred use in patients who frequently consume staining beverages, potentially extending the aesthetic lifespan of restorations and reducing the need for maintenance or replacement [6,11,12]. Additionally, these findings emphasize the need for patient education about the staining potential of common dietary substances and the adoption of strict oral hygiene practices to maintain restoration integrity [3,12,13]. Different types of other beverages can be used for future research based on these findings, assessing the usefulness of surface sealants in long-term clinical conditions [14,15]. Furthermore, different studies on molecular interactions between staining compounds and resin matrix components could create enhanced color stability restorative materials [1,6,13].

Limitation

This study has several limitations. First, laboratory conditions do not replicate dynamic oral factors, including salivary protective factors, temperature fluctuations, complex pellicle formation, and oral microbiome interactions. Second, the continuous 30-day immersion protocol does not precisely mirror intermittent and real-world human beverage consumption. Third, the specimens did not undergo simulated mechanical wear or toothbrushing cycles during the testing period, which can alter surface roughness and discoloration over time. In order to perform future research, dynamic aging models should be applied with mechanical brushing simulation. It should evaluate diverse baseline composite shades and track beverage pH drops to better generalize these findings to clinical practice.

## Conclusions

In conclusion, this study offers comprehensive, clinically relevant evidence on the color stability of hybrid and nano-hybrid resin composites, emphasizing the importance of material selection and patient management in restorative dentistry. The insights gained here contribute to optimizing restorative protocols aimed at improving the aesthetic outcomes and longevity of resin composite restorations in modern dental practice.
